# Coupling between the Circadian Clock and Cell Cycle Oscillators: Implication for Healthy Cells and Malignant Growth

**DOI:** 10.3389/fneur.2015.00096

**Published:** 2015-05-11

**Authors:** Celine Feillet, Gijsbertus T. J. van der Horst, Francis Levi, David A. Rand, Franck Delaunay

**Affiliations:** ^1^Circadian Systems Biology, CNRS, INSERM, Institut de Biologie Valrose, Université Nice Sophia Antipolis, Nice, France; ^2^Department of Genetics, Erasmus University Medical Center, Rotterdam, Netherlands; ^3^Cancer Chronotherapy Unit, University of Warwick, Coventry, UK; ^4^Systems Biology Centre, University of Warwick, Coventry, UK

**Keywords:** circadian desynchrony, cell cycle, coupling, cancer, mathematical modeling

## Abstract

Uncontrolled cell proliferation is one of the key features leading to cancer. Seminal works in chronobiology have revealed that disruption of the circadian timing system in mice, either by surgical, genetic, or environmental manipulation, increased tumor development. In humans, shift work is a risk factor for cancer. Based on these observations, the link between the circadian clock and cell cycle has become intuitive. But despite identification of molecular connections between the two processes, the influence of the clock on the dynamics of the cell cycle has never been formally observed. Recently, two studies combining single live cell imaging with computational methods have shed light on robust coupling between clock and cell cycle oscillators. We recapitulate here these novel findings and integrate them with earlier results in both healthy and cancerous cells. Moreover, we propose that the cell cycle may be synchronized or slowed down through coupling with the circadian clock, which results in reduced tumor growth. More than ever, systems biology has become instrumental to understand the dynamic interaction between the circadian clock and cell cycle, which is critical in cellular coordination and for diseases such as cancer.

## Clock and Cell Cycle, 2 Biological Oscillators

### The circadian clock

The Earth’s rotation results in predictable daily variations of environmental conditions (light/dark, food, temperature). Biological clocks give an unbiased estimation of time and allow coordination of physiology in anticipation to recurring changes.

Circadian clocks are implicated in normal functioning of numerous systems (digestive, endocrine, cellular). They control 24-h rhythms, termed as “circadian rhythms” (sleep, body temperature, cortisol secretion …).

An important milestone in the field was the discovery of the mammalian circadian “conductor”: the suprachiasmatic nuclei of the hypothalamus (SCN) (1, 2). From the molecular standpoint, the SCN machinery consists of feedback loops relying on clock genes. A main loop involves CLOCK and BMAL1, which heterodimerize and activate the transcription of *Period* (*Per1–2–3*) and *Cryptochrome* (*Cry1–2*) genes. PER:CRY proteins in turn inhibit their own transcription by direct interaction with CLOCK:BMAL1. This essential loop is modulated by the REV-ERBα–β and RORα–β–γ proteins, which operate a negative and positive feedback, respectively, on *Bmal1* transcription. This genetic network is also extensively regulated via post-translational processes. The molecular clockwork acts autonomously and, in the absence of resetting cues, oscillates with a period close to 24 h; it is the basis of circadian rhythmicity and defines the endogenous period of a clock (3). It is synchronized to a sharp 24-h period by environmental parameters (light/dark, feeding cycles, hormones). A rhythmic message integrating environmental informations is then generated by the SCN, and redistributed to the entire organism to synchronize physiological functions. Circadian rhythmicity and clock genes are not an exclusive property of the SCN as clock genes are rhythmically expressed in nearly all cells. Each individual cell can thus be regarded as a circadian clock (4, 5).

### The cell cycle

Cell cycle is the process leading to cell division. It consists of two critical phases: the S phase, in which the cell replicates its DNA, and the M phase where it divides (mitosis); they are preceded by growth phases G1 and G2, respectively. Non-dividing (somatic) cells are in a quiescent state (G0). They may resume cell division, depending on environmental parameters such as growth factors, to enter G1 phase (6, 7).

Progression of the cell cycle relies on transient and sequential activation of cyclin-dependent kinases (CDKs) forming complexes with cyclins (CCN). Cell cycle successively depends on CCND/CDK4–6 (G1), CCNE/CDK2 (G1/S transition), CCNA/CDK2 (S), CCNA/CDK1 (S/G2 transition), and CCNB/CDK1 [M – (8–10)]. Activity of these enzymatic complexes finely tunes cell cycle duration, especially at critical checkpoints. Association with CDK inhibitors (CKI–P16, P27, P21) or phosphorylation by the kinase WEE1, inhibit activity of targeted CCN/CDK across the cycle. On the other hand, they are activated by phosphatases such as CDC25A–B–C (11). These activators or inhibitors are targets of proteins involved in DNA repair. Indeed, a double-strand DNA break activates the ataxia telangiectasia mutated (ATM) and check-point kinase 2 (CHK2) proteins, while a single-strand break or a replication error activate ataxia telangiectasia related (ATR) and check-point kinase 1 (CHK1) proteins (12, 13). These complexes cause a cell cycle arrest by indirect induction of CKI.

From a dynamical point of view, the cell cycle can be modeled either as a series of checkpoints (“domino” model) or as a biochemical oscillator where successive waves of CCN/CDK activity control its progression (14–16). In the following parts, we will use this second model as it recapitulates the rhythmic properties of the cell cycle.

Both cell cycle and molecular clocks display periodic phases of activation and repression from transcriptional to post-translational levels (17). Thus, they can be considered as biological oscillators coexisting in dividing cells.

## The Circadian Gating Model

In unicellular organisms, circadian rhythms and cell division are considered as non-independent processes. In particular, the circadian system controls timing of cell division both in prokaryotic and eukaryotic species. This is the case in the cyanobacterium *Synechococcus elongatus* and in the flagellate alga *Euglena gracilis*, where the molecular clock operates as a “gating” and only allows cell division at specific circadian phases (18–20). Thus, the circadian oscillator can be viewed as an additional checkpoint for mitosis.

It was tempting to extrapolate this phenomenon to other organisms; although some research was conducted toward demonstration of circadian gating of the cell cycle in mammalian cells, results led to controversial evidence. While a study from 2004 reported that the majority of cell divisions occur in three phases of the circadian cycle (4), two more recent papers describe an absence of cell cycle regulation by the circadian clock (21, 22).

## Dynamical Coupling between Clock and Cell Cycle

**Figure 1:**
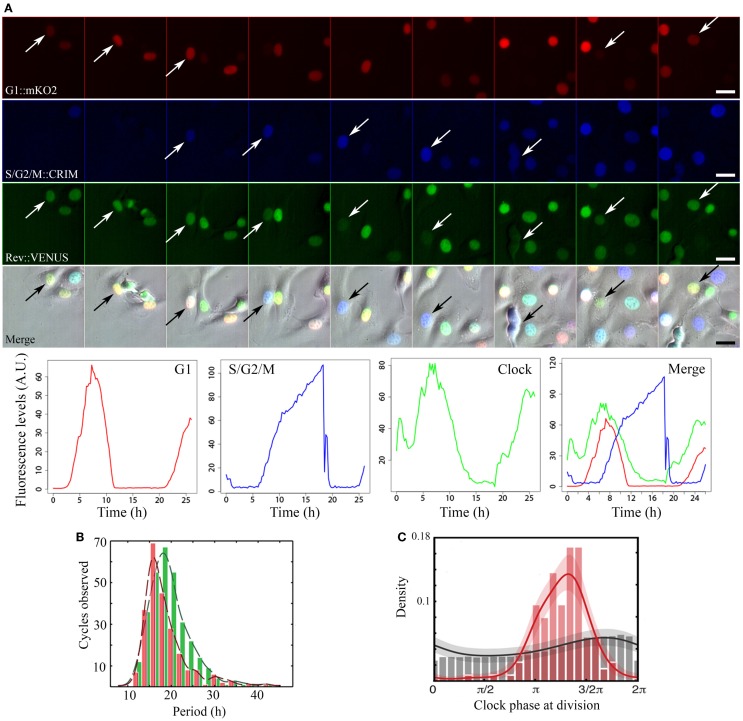
**Image acquisition, fluorescence quantification, period, and phase dynamics in non-synchronized NIH3T3-REVERBα:VENUS_FUCCI cells**. **(A)** Time series of a representative single cycle for the various fluorescent reporters and respective quantified traces: from top to bottom: Cell Cycle_G1 (red) = CDT1:mKO2, Cell Cycle_S/G2/M (blue) = GEMININ:E2CRIMSON, Circadian Clock (green) = REVERBα:VENUS, Merge = fluorescent channels combined with the corresponding brightfield image. Arrows point to tracked cell nuclei. Images are 2.5 h apart. Traces at the bottom have been plotted from measured intensities extracted from tracking with the LineageTracker plugin for ImageJ. **(B)** Histograms showing distribution of periods for both the clock (green) and cell cycle (red) in the whole population. In non-synchronized cells, mean clock period (19.4 ± 0.5 h) is not significantly different from mean cell cycle period: (18.6 ± 0.6 h). **(C)** Phase histograms for the same cells. Gray histogram and trace show random background densities. Colored histogram and trace show the observed phase of the clock at division. We observe a preferred clock phase for performing cell division (phase locking).

Faced with these contradictions, we and others have recently investigated the circadian clock-cell cycle connection by quantifying the dynamics of the two oscillators in real time, in single live mammalian cells (23, 24). Both studies used the circadian clock reporter REV-ERBα:VENUS (4). For cell cycle, Bieler et al. scored timing of division. We added a cell cycle reporter system [fluorescent ubiquitination-based cell cycle indicator, FUCCI; (25)] probing cell cycle progression. These fluorescent markers were used to quantitatively determine the properties of each oscillator in single NIH3T3 cells. Timelapse imaging combined to extensive statistical analysis and modeling exposed the dynamical properties of these biological oscillators (Figure 1A).

In cells where neither clock nor cell cycle was synchronized by external cues, they appear robustly coupled, with a 1:1 ratio between their respective periods, over a wide range of durations (18–27 h; Figures 1B,C). A clear shortening of the circadian period occurred in dividing cells compared to non-dividing cells thus revealing an influence of cell cycle on the clock. Mathematical analysis and stochastic modeling unambiguously showed that coupling rather than gating governs cell cycle and circadian interaction in NIH3T3 cells. It also revealed that the clock reporter reproducibly peaked about 5 h after cell division. This feature is termed as phase locking (Figure 1C). Changing cell cycle duration impacted on circadian cycles, but 1:1 locking was resilient to such changes (23, 24). Additionally, inhibition of the cell cycle at the G1/S or G2/M transitions lengthened circadian intervals and delayed division phase. Bieler et al. looked at the reverse interaction by changing circadian period. This did not affect cell cycle length but advanced division with respect to circadian phase. The authors thus proposed a unidirectional coupling from the cell cycle to the circadian clock (23).

The above experiments were performed in absence of external cues. However, *in vivo* cellular clocks are subjected to synchronizing messages (e.g., corticosteroids, temperature). We therefore investigated coupling after a 2 h treatment with dexamethasone, which resets the circadian clock. We observed two distinct dynamical behaviors coexisting within the cell population. Whereas one sub-population kept a 1:1 phase locking, the ratio of cell cycle and clock periods was fixed to 3:2 in the other one (i.e., 3 cell cycles for 2 clock cycles). Moreover, when projecting the timing of mitosis across the whole experiment, we observed a clear clustering of cell division suggesting that the cell cycle was synchronized by physiological cues via the circadian clock. We thus inferred a bidirectional coupling between the two oscillators, supported by mathematical modeling (24).

Although the team of Naef concluded to a unidirectional coupling whereas we concluded to a bidirectional coupling, both studies led us to reject the concept of “gating” of the cell cycle by the clock in mammalian cells, usually put forward without theoretical support (23, 24). In the end, it seems that the cell cycle is capable of impacting on the circadian clock and vice versa, the dominant influence being dependent on the environment of the cell.

## Molecular Coupling between Clock and Cell Cycle

Molecular mechanisms underlying progression of the clock and cell cycle have been extensively studied. Yet, little is known about candidates that could underlie coupling between the two oscillators.

### Influence from the circadian clock on the cell cycle

The molecular clock impacts on the cell cycle, by transcriptional control or direct protein–protein interactions. For instance, in G1, the CKI P21 is transcriptionally regulated by REV-ERBα and RORα/γ (26). At G1/S transition, NONO regulates the p16-Ink4A check-point gene in a PER-dependent fashion (27). Transcription of the WEE1 kinase (G2/M transition) is tightly controlled by the CLOCK:BMAL1 dimer (28).

At the post-translational level, CRY modulates CHK1/ATR (G1/S transition checkpoint) by interacting with TIMELESS (TIM) in a time-of-day-dependent manner. PER and TIM also regulate the G2/M transition via interactions with CHK2-ATM (12, 13, 29, 30).

Other clock-controlled cell cycle regulators include known oncogenes (*c-Myc*, *Mdm2*, and β-*catenin*), cyclins (CCND1, B, and A), and tumor suppressor *p53*. Many key cell cycle regulators, such as *Cdk4*, *Itga6*, *Wnt3*, *LHx2*, *Tcf4*, *Sox 9*, and *Smad7* are also directly clock-regulated (31). This multitude of interactions gives a reasonable explanation why loss of coupling between clock and cell cycle may play a key role in carcinogenesis and abnormal growth *in vivo* (see below).

### Influence of the cell cycle on the circadian clock

Apart from transcription silencing occurring at mitosis (32), which is bound to influence the circadian feedback loops, molecular evidence of cell cycle-dependent control of the circadian clock is sparse. Nevertheless, DNA damage can phase advance cellular and behavioral circadian rhythms in a dose- and time-dependent manner. The underlying mechanism involves ATM-mediated damage signaling, possibly through interaction with the PER and TIM proteins (33).

More recently, an impact of the tumor suppressors P53 and promyelocytic leukemia (PML) proteins on circadian function was proposed: *Per2* transcription is repressed by P53, which prevents binding of CLOCK:BMAL1. At the post translational level, PML physically interacts with PER2, and promotes its nuclear localization. These molecular connections are translated into altered circadian behavior (34, 35). As a whole, these results point to a global influence of cell cycle regulation on circadian function.

Among these molecular interactions, only *p53* has been found to impact on clock and cell cycle in a bidirectional way. Thus, it may participate in coupling between the two oscillators, which remains to be confirmed.

## Cellular Consequences of Coupling in Healthy Cells

A major impact of clock and cell cycle coupling on cell physiology resides in timed mitoses. For instance, about 1/6th of human epidermal cells divide daily, mainly at night (36), which is determined by intrinsic *Bmal1* expression (37). Rhythmicity in cell cycle parameters was also found in the hematopoietic and immune systems (spleen, thymus, bone marrow), gastro-intestinal tract (colon, liver), skin, and cornea of rodents and/or humans (38). DNA synthesis and mitosis rhythmicity seem impervious to ablation of adrenals, medulla, pituitary, or even SCN, although sometimes displaying altered characteristics (38–40). Hence, if systemic circadian cues are required to coordinate cell divisions in the whole organism, local clock/cell cycle coupling likely governs rhythmic mitosis at the cellular and tissue levels.

In line with this hypothesis, different populations of epidermal stem cells were found to express clock genes in opposite phases, which results in a differential propensity for activation. Specific disruption of the circadian clock in these cells led to premature epidermal aging, which confirms that local coupling is necessary to ensure tissue integrity (41).

Coupling even manifests itself in a post-mitotic tissue such as the adult brain, where quiescent neural progenitors in the hippocampus show rhythmic proliferation. *Per2* and *Bmal1* play a critical role in this rhythmic neurogenesis, impacting on cognitive function. Mathematical modeling pointed to clock-driven *p21* expression as a trigger for cell cycle progression through regulation of the CCND/CDK4–6 complexes (42).

In the adult liver, only 1% of hepatocytes show rhythmic mitoses under normal conditions. After partial hepatectomy, however, most of the remaining hepatocytes enter cell cycle synchronously, in a clock-dependent manner, which will restore liver mass within few days. In arrhythmic *Cry* deficient mice, liver regeneration still occurs, although full recovery of liver mass is delayed (28). A more common situation requiring controlled cell division at an adult age is wound repair: after skin incision, wound healing defects were found in mice lacking NONO, a possible link between clock and cell cycle. (27).

Thus, crosstalk between the clock and cell cycle oscillators is required for timed and optimal organization of cell proliferation both in healthy tissue and in times of challenge such as regeneration.

## Importance of Coupling in Times of Challenge: From Healthy Cells to Pathology

A major interest of the interplay between the clock and cell cycle is that a dysfunction of either system can lead to diseases such as cancer. Among the hallmarks of cancer, genome instability and mutations in cell cycle genes are a recurring enabling factor (43). Indeed, mutation in *Cyclin*, *Cdk*, or *Cki* genes was found in 90% of human cancers (44). However, evidence is increasing that cancer cells also display a deregulation of the circadian clockwork, which may promote abnormal proliferation (31).

### Circadian disruption and cell cycle deregulation

Although mutations in clock genes are frequent in human cancer cells, it is unclear whether it may be a primary cause of cancer development. *Per2* mutant mice display tumor-prone phenotypes and deregulation of various cyclins, proto-oncogenes, and tumor suppressors (45). On the other hand, *Cry1*/*Cry2* KO mice do not show increased cancer development after γ-irradiation (46). Thus, deregulation of the core clock cannot fully account for the observed phenotype in *Per2* mutant mice. Additionally, mutation of *Bmal1* or *Clock* does not lead to enhanced cancer development (47, 48). These results are subject to debate, as a more recent study showed that *Per1*^−/−^*Per2*^−/−^, *Cry1*^−/−^*Cry2*^−/−^, and *Bmal1*^−/+^ mice have increased spontaneous and radiation-induced tumor development (49). So it remains unclear whether a mutation in clock genes is by itself sufficient to trigger cancer development. These observed cancer-prone phenotypes might be due to a non-circadian function of these genes.

If clock gene mutations do not induce cancer *per se*, systemic environmental disruption of the circadian function may impact on cancer development and cell proliferation. Constant light exposure or ablation of the pineal gland results in perturbation of endocrine rhythms and increase carcinogenesis in liver and mammary glands of rodents (50, 51). In line with these results, circadian disruption following SCN lesions or chronic jetlag increases proliferation of implanted Glasgow osteosarcoma or pancreatic adenocarcinoma (52, 53). Chronic jetlag also promotes tumor development in the liver of mice exposed to the hepatic carcinogen diethylnitrosamine (54).

The latter finding is of particular interest when considering cancer development in humans. As already mentioned, circadian alterations at the molecular level have been found in numerous human cancers. At the epidemiological level, studies involving shift workers have associated circadian disruption with an increased risk of cancer development (31). Since 2007, the International Agency for Research on Cancer even listed “shiftwork that involves circadian disruption” as a “probable carcinogen.”

### Cell cycle deregulation and cancer lead to circadian disruption

Many human cancer cell lines (including breast, prostate, colon, liver, and lung), which by definition show abnormal cell cycle progression and proliferation, also display abnormal circadian gene expression (31). In the context of bidirectional interaction between the clock and cell cycle oscillators, it is tricky to decipher which alteration is the cause or consequence. If mutations in cell cycle genes are the primary element, it will in turn impact on the circadian clock. Another possibility is that cell cycle and circadian functions will be synergistically disrupted as a consequence of their coupling.

At the physiological level, recent studies have explored circadian rhythmicity in metastatic colorectal or breast cancer patients (rest activity and/or salivary cortisol rhythms). All studies concluded to large disparities in behavioral and endocrine rhythmicity in these patients, ranging from robust to absent rhythms. Interestingly, the outcome of the disease in patients with damped or abnormal circadian rhythmicity is generally unfavorable, independently of other prognostic factors (38).

### Circadian lifestyle and slowing down tumor progression: Perspectives of coupling

Temporal restricted feeding (RF), also known as meal timing, powerfully entrains peripheral clocks (55). It is also capable of restoring circadian rhythms in peripheral tissues of otherwise arrhythmic mice (56). Tumor cells often show abnormal circadian rhythms in LD and *ad libitum* feeding conditions, but they are not blind to cyclic metabolic cues. Two studies tested the impact of RF on growth rate of implanted Glasgow osteosarcoma or P03 pancreatic adenocarcinoma in mice (57, 58). At the physiological level, this treatment increased the amplitude of body temperature rhythms, known to be a powerful circadian synchronizer (59, 60). Interestingly, tumor size was significantly reduced and survival was prolonged in mice submitted to RF. Thus, reinforcement of host’s circadian rhythms may lead to improved host-mediated tumor control or alteration of the tumor circadian clock, which in the end slows down tumor progression.

Looking at this data with the newly discovered bidirectional coupling between clock and cell cycle, we would like to put forward the possibility that the cell cycle may be synchronized/slowed down through coupling with the circadian clock. Hence, it would be possible to use a known circadian synchronizer such as timed feeding or high-amplitude temperature cycles to re-entrain or reinforce the circadian clock in tumor cells. These cells tend to proliferate at a high rate, with periods shorter than 24 h. Entraining the circadian clock in these cells could slow down cell cycle, through coupling between the two oscillators. This would, in the end, delay tumor progression.

## Conclusion and Perspectives

In mammals, the circadian and cell cycle oscillators were long considered as two completely independent entities, or were at best regarded as a circadian clock gating cell cycle progression. These views are seriously challenged by the recently established bidirectionality of clock and cell cycle interactions. At the moment, only P53 has been identified as capable of impinging on both clock and cell cycle, but its role in coupling needs to be confirmed. Given the complexity of these oscillatory circuits, additional candidates likely exist, but remain to be found.

In terms of physiology, the consequences of coupling in healthy cells are obvious. But coupling becomes critical when considering a pathology such as cancer, where circadian and cell cycle phenomena are intertwined. The benefits of chronotherapeutics for cancer treatment is well established (38). They are a consequence of circadian fluctuations in toxicity and efficacy of cancer drugs (and drug metabolism in general). Considering that reinforcing the circadian clock through a change in lifestyle (e.g., meal timing) is capable of slowing down tumor progression, probably through coupling with the cell cycle, we may be able to propose individualized life schedules that will potentiate the effect of chronotherapy, to reduce tumor progression without additional pharmacological intervention. Not to say that reinforcing circadian timing will also alleviate dampening of circadian rhythmicity, which is often a complaint from cancer patients. This long-term perspective may have a high impact on human health.

## Conflict of Interest Statement

The authors declare that the research was conducted in the absence of any commercial or financial relationships that could be construed as a potential conflict of interest.
